# Preoperative use of angiotensin-converting enzyme inhibitors, angiotensin II receptor blockers and diuretics increases the risk of dehydration after ileostomy formation: population-based cohort study

**DOI:** 10.1093/bjsopen/zrae051

**Published:** 2024-05-31

**Authors:** Louise de la Motte, Caroline Nordenvall, Anna Martling, Christian Buchli

**Affiliations:** Department of Molecular Medicine and Surgery, Karolinska Institutet, Stockholm, Sweden; Department of Pelvic Cancer, Colorectal Surgery Unit, Karolinska University Hospital, Stockholm, Sweden; Department of Molecular Medicine and Surgery, Karolinska Institutet, Stockholm, Sweden; Department of Pelvic Cancer, Colorectal Surgery Unit, Karolinska University Hospital, Stockholm, Sweden; Department of Molecular Medicine and Surgery, Karolinska Institutet, Stockholm, Sweden; Department of Pelvic Cancer, Colorectal Surgery Unit, Karolinska University Hospital, Stockholm, Sweden; Department of Molecular Medicine and Surgery, Karolinska Institutet, Stockholm, Sweden; Department of Pelvic Cancer, Colorectal Surgery Unit, Karolinska University Hospital, Stockholm, Sweden

## Abstract

**Background:**

Readmission rates following ileostomy formation are high. Dehydration and consecutive renal failure are common causes of readmission, potentially pronounced by drugs affecting the homeostasis. The aim of the study was to assess the risk of dehydration after ileostomy formation in patients treated with angiotensin-converting enzyme inhibitors (ACEI), angiotensin II receptor blockers (ARB) or diuretics.

**Method:**

This nationwide population-based cohort study used data derived from the Colorectal Cancer Data Base of several Swedish healthcare registers. The study included all patients operated on with elective anterior resection and temporary ileostomy for rectal cancer clinically staged I–III in Sweden in 2007–2016. Exposure was at least two dispensations of ACEI, ARB or diuretics within 1 year prior to surgery. Outcome was 90-day readmission due to dehydration including acute renal failure.

**Results:**

In total, 3252 patients were included with 1173 (36.1%) exposed to ACEI, ARB or diuretics. The cumulative incidence for 90-day readmission due to dehydration was 29.0% (151 of 520) for exposed *versus* 13.8% (98 of 712) for unexposed. The proportion of readmissions due to any reason was 44.3% (520 of 1173) for exposed compared to 34.2% (712 of 2079) for unexposed. The incidence rate ratio for readmission due to dehydration was 2.83 (95% c.i. 2.21 to 3.63, *P* < 0.001). The hazard rate ratio was 2.45 (95% c.i. 1.83 to 3.27, *P* < 0.001) after adjusting for age, gender and comorbidity.

**Conclusion:**

Medication with ACEI, ARB or diuretics defines a vulnerable patient group with increased risk of readmission due to dehydration after ileostomy formation.

## Teaser

This is the first population-based cohort study investigating the association between preoperative use of ACEI, ARB and diuretics on postoperative dehydration and acute renal failure in Swedish rectal cancer patients operated with anterior resection and temporary ileostomy. A two- to three-fold risk of 90-day readmission was observed among exposed participants compared to unexposed, with the risk being most pronounced for ACEI users.

## Introduction

Protection of colorectal anastomoses, de-functioning of inflamed or obstructed colon and acute colectomy are common reasons to perform ileostomies. Absence of colonic passage may result in high stoma output with loss of fluids and electrolytes. Readmission rates of 20% within 30 days following ileostomy formation have been reported, with dehydration being the most common cause of readmission occurring in 6% of patients^1^. Consecutive negative impact on renal function has also been reported in the early postoperative phase and may persist after stoma closure, indicating risk of long-term renal impairment^2–8^. Being able to identify patients at risk of dehydration is therefore of great importance in order to prevent unplanned readmissions and protect long-term health after ileostomy formation^9^.

Medication with angiotensin-converting enzyme inhibitors (ACEI), angiotensin II receptor blockers (ARB) and diuretics are today commonly used in patients with hypertension or cardiovascular disease and are known to cause renal impairment in dehydrated patients. In Sweden, more than 2 million people aged 20 or above had at least one prescription of ARB, ACEI or diuretics during 2021^10^. Usage of ACEI, ARB or diuretics in patients with ileostomy is therefore not uncommon. A few observational studies have previously reported ACEI, ARB and diuretics being risk factors for readmission due to dehydration and renal impairment following ileostomy formation^2,11,12^, but to our knowledge, larger population-based studies are lacking. Medication is usually interrupted the day of surgery, but the postoperative handling is less clear. At present, there are no official recommendations for treatment with ACEI, ARB and diuretics in patients with ileostomies.

The aim of this nationwide cohort study was therefore to investigate if preoperative use of ACEI, ARB or diuretics increases the risk of dehydration after elective ileostomy formation.

## Methods

### Study design

This population-based cohort study investigates the effect of preoperative use of ACEI, ARB or diuretics on the risk of readmission due to dehydration in rectal cancer patients treated with an ileostomy to protect the colorectal anastomosis after anterior resection. Data are derived from the Colorectal Cancer Data Base (CRCBaSe), a mega-linkage of several Swedish nationwide registers^13^. The study was approved by the Regional Ethical Review Board (Dnr 2014/71-31, 2018/328-32 and 2021-00342). This article was written according to the STROBE checklist for reporting of observational cohort studies^14^.

### The Colorectal Cancer Data Base

The CRCBaSe is based on the Swedish Colorectal Cancer Register (SCRCR), a national quality register including nearly all diagnosed with rectal and colon cancers in Sweden since 1995–2007^15^. The personal number, a unique identifier of all Swedish residents, enables linkage of data from several official registers held at the National Board of Health and Welfare (including the Prescribed Drug Register, the Inpatient Register, the Cause of Death Register) and Statistics Sweden^13^. At the time of data extraction for this study, CRCBaSe contained data of approximately 2.4 million individuals, including patients with colorectal cancer, comparators and relatives^13^.

### Participants

**Fig. 1:**
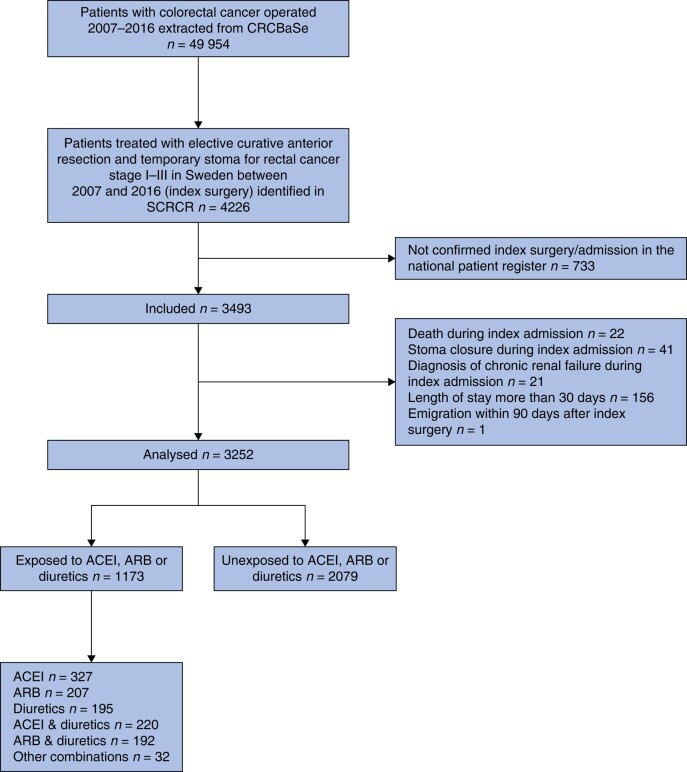
Flowchart illustrating selection of study participants ACEI, angiotensin-converting enzyme inhibitors; ARB, angiotensin II receptor blockers; CRCBaSe, Colorectal Cancer Data Base.

All patients 18 years and older at the time of diagnosis treated with elective anterior resection and temporary ileostomy with curative intent for rectal cancer clinically staged I–III (index surgery) in Sweden between 1 January 2007 and 31 December 2016 were considered eligible. In the event of synchronous tumours, the one with the highest clinical tumour stage was included. Index admission started the day of admission prior to index surgery and was the sum of uninterrupted admissions generating posts in the Inpatient Register. Index admissions without confirmed procedure codes for anterior resection and ileostomy in the Inpatient Register were excluded. Other exclusion criteria included systemic disease or ileostomy prior to index surgery, diagnosis of chronic renal failure or stoma closure or death during index admission and length of index admission exceeding 30 days. Emigration within 90 days from index surgery was also an exclusion criterion due to loss of follow-up (*Fig. 1* and *Supplementary Appendix S1*).

### Pilot study

The present nationwide cohort study is based on a pilot study investigating the risk of readmission due to dehydration or acute renal failure in patients treated with ACEI or ARB. Medical records of 749 patients with rectal cancer operated with anterior resection and temporary ileostomy in the Stockholm Region 2007–2015 were reviewed with similar inclusion and exclusion criteria. Patients treated with diuretics, no ACEI/ARB, were excluded in the analysis (*n* = 31). Exposure to ACEI/ARB were based on the same ATC codes as the main study but defined as regular prescription at the day of discharge after index operation. Outcome was death or readmission due to dehydration or acute renal failure within 90 days after index operation, based on the same ICD-10 codes as the main study or predefined notes identified in medical records at readmission. Comparison on an individual level between data derived from CRCBaSe and medical records was not possible as CRCBaSe data are pseudo-anonymized; however the results of the pilot study were used to compare effect sizes.

### Exposure: dispensation of medications

Participants with at least two dispensations of ACEI, ARB or diuretics at a Swedish pharmacy within a time period of 365 days prior to index surgery were considered exposed. This information was obtained from the Prescribed Drug Register based on Anatomical Therapeutical Chemical (ATC) codes. Exposed participants were classified as either ACEI (only), ARB (only), diuretics (only), ACEI and diuretics (combined), ARB and diuretics (combined) or other combinations (*Supplementary Appendix S2*). To assess differences in the risk of dehydration between substances, a subgroup analysis was performed comparing the exposed subgroups with the unexposed group separately. Participants in the other combinations group were not included in the subgroup analysis.

### Outcome of interest: readmission due to dehydration

Readmission to hospital within 90 days after index surgery with diagnosis of dehydration was the measured outcome, with time at risk starting the day after discharge from index admission. Study participants were censored in case of readmission due to other reasons, death or at end of follow-up 90 days after index surgery. Diagnosis of dehydration was based on the International Classification of Disease codes 10th edition (ICD-10) for volume depletion, electrolyte imbalance or acute renal failure. ICD-10 codes and admission and discharge dates of inpatient care were collected from the Inpatient Register and dates of death from the Cause of Death Register (*Supplementary Appendix S3*).

### Additional variables

Patient, tumour and treatment characteristics were extracted from SCRCR and Statistics Sweden including gender, age at cancer diagnosis and ASA classification (*Supplementary Appendix S4*). BMI was calculated according to the formula [weight (kg)]/[length (m)]^216^ and tumours classified according to TNM stage. Length of hospital stay was calculated based on number of days between index surgery and discharge of index admission. Dispensations of calcium channel blockers or beta blockers (at least two within a time period of 365 days prior to index surgery), as well as dispensation of loperamide, opioids or oxycodone/naloxone (at least one after discharge from index surgery but before readmission/end of follow-up 90 days), were collected based on ATC codes from the Prescribed Drug Register. The Charlson Comorbidity Index (CCI) score (weighted) was calculated based on ICD-10 codes from the Inpatient Register (5 years prior to colorectal cancer diagnosis) and Swedish Cancer Registry (10 years prior to colorectal cancer diagnosis)^17^. No point was given for colorectal cancer. Study participants with ICD-10 codes I00–99 during index admission were regarded as having cardiovascular comorbidity and those with ICD-10 codes E10–14 as having diabetes mellitus^18^.

### Statistical methods

Statistical analyses were performed with Stata version 15 (StataCorp LP, College Station, TX). Wilcoxon rank sum or Fisher exact tests were used for group comparisons with a two-sided 0.05 level of statistical significance. The time to readmission was compared between groups by incidence rate ratios (IRR) and the Kaplan–Meier method including log rank test. After graphical assessment of the proportional hazard assumption by ‘log-log’ plots and Schoenfeld residuals, Cox regression models were used to adjust analyses for age, gender, ASA score and CCI score (weighted). Further covariates such as country of birth, BMI, preoperative radiotherapy, duration of index surgery, perioperative bleeding, length of stay during index admission, dispensation of loperamide, opioids or oxycodone/naloxone and pathological TNM stage were considered for inclusion in final models if they changed point estimates by more than 10% or if the interaction term with the group variable indicated significant effect modification. Participants with no dispensations registered during the studied time period were considered healthy/unmedicated and were allocated to the unexposed group. Readmissions with no ICD-10 codes registered were included in the *any reason group* because the reason for readmission was unknown.

## Results

### Participants

Between 2007 and 2016, 4226 elective anterior resections with temporary stoma for rectal cancer clinically staged I–III were performed in Sweden. Seven hundred and thirty-three patients were excluded due to admissions without confirmed procedure codes for anterior resection and ileostomy in the Inpatient Register; 3252 patients were included in the analysis (*Fig. 1*), including 9 patients with synchronous tumours. In total, 36.1% (1173 of 3252) had at least two dispensations of ACEI, ARB or diuretics within 365 days prior to index surgery. Overall, 2079 participants were defined as unexposed.

Patient characteristics at baseline and information regarding oncological treatment are presented in *Table 1*. The proportion of men and age at diagnosis was higher in the exposed group compared to the unexposed group (66.2% *versus* 57.8% and 70 years *versus* 65 years respectively). The exposed group also had higher ASA and CCI scores, with a diagnosis of cardiovascular disease, diabetes and obesity (BMI > 30) being more common. Preoperative radiotherapy and postoperative chemotherapy were more often used in the unexposed group (68.9% *versus* 62.8% and 27.9% *versus* 20.8% respectively). The observed differences in median perioperative bleeding and postoperative time during index admission were of limited clinical significance.

**Table 1:** Patient and treatment characteristics of 3252 Swedish patients treated with anterior resection and ileostomy for rectal cancer between 2007 and 2016

	Exposed *N* = 1173	Unexposed *N* = 2079	*P*
**Sex**			<0.001
Male	776 (66.2)	1201 (57.8)	
Female	397 (33.8)	878 (42.2)	
**Country of birth**			0.801
Sweden	993 (84.7)	1751(84.2)	
Other	180 (15.4)	327 (15.7)	
Missing	0 (0.0)	1 (0.1)	
Age at cancer diagnosis (years)	70 (64–75)	65 (57–71)	<0.001
**Age (years)**			<0.001
<55	58 (4.9)	381 (18.3)	
55–65	249 (21.2)	609 (29.3)	
65–75	549 (46.8)	799 (38.4)	
>75	317 (27.0)	290 (14.0)	
BMI (kg/m^2^)	26.3 (24.2–29.2)	25.2 (23.0–27.7)	<0.001
**BMI (kg/m^2^)**			<0.001
<18.5	11 (0.9)	30 (1.4)	
18.5–24.9	369 (31.5)	914 (44.0)	
25.0–29.9	508 (43.3)	811 (39.0)	
>30	237 (20.2)	248 (11.9)	
Missing	48 (4.1)	76 (3.7)	
**ASA classification**			<0.001
ASA 1	39 (3.3)	803 (38.6)	
ASA 2	826 (70.4)	1062 (51.1)	
ASA 3 + 4	296 (25.2)	179 (8.6)	
Missing	12 (1.0)	35 (1.7)	
**CCI score**			<0.001
0	738 (62.9)	1670 (80.3)	
1	164 (14.0)	113 (5.4)	
2	169 (14.4)	234 (11.3)	
>=3	92 (7.8)	45 (2.2)	
Missing	10 (0.9)	17 (0.8)	
**Diagnosis of cardiovascular disease**			<0.001
No	382 (32.6)	1796 (86.4)	
Yes	791 (67.4)	283 (13.6)	
**Diagnosis of diabetes mellitus**			<0.001
No	976 (83.2)	1985 (95.5)	
Yes	197 (16.8)	94 (4.5)	
**Preoperative radiotherapy**			<0.001
No	437 (37.3)	646 (31.1)	
Yes	736 (62.8)	1433 (68.9)	
Length of index surgery (min)	257 (206–240)	254 (200–326)	0.057
Perioperative bleeding index surgery (ml)	380 (200–650)	300 (150–600)	<0.001
Time from index surgery to discharge of index admission (days)	10 (7–15)	9 (7–14)	<0.001
**Pathological TNM stage**			0.466
1	398 (33.9)	682 (32.8)	
2	347 (29.6)	584 (28.1)	
3	420 (35.8)	801 (38.5)	
4	6 (0.5)	9 (0.4)	
Missing	2 (0.2)	3 (0.1)	
**Postoperative chemotherapy**			<0.001
No	929 (79.2)	1499 (72.1)	
Yes	244 (20.8)	580 (27.9)	

Continuous variables reported as median (i.q.r.) and categorical variables as frequency (proportion). Exposed defined as having at least two dispensations of ACEI, ARB or diuretics within a time period of 365 days prior to index surgery. *P* calculated using Fisher’s exact test or Wilcoxon rank sum-test. ACEI, angiotensin-converting enzyme inhibitors; ARB, angiotensin II receptor blockers; CCI, Charlson Comorbidity Index (weighted).

### Dispensation of medications

One hundred and forty-two patients had no dispensation registered. Among the exposed (1173), the distribution of dispensations was 27.9% ACEI only, 17.7% ARB only, 16.6% diuretics only, 18.8% ACEI and diuretics combined, 16.3% ARB and diuretics combined and 2.7% other combinations (*Fig. 1*). Dispensation of calcium channel blockers and beta blockers was higher in the exposed group (31.0% *versus* 8.2% and 45.2% *versus* 12.2% respectively), whereas dispensation of opioids was higher in the unexposed group (51.3% *versus* 45.4%). Dispensation of loperamide and oxycodone/naloxone was similar in both groups.

### Readmissions within 90 days after index surgery

In total, 37.9% (1232 of 3252) were readmitted at least once within 90 days after index surgery. In 15 cases no ICD-10 codes were registered for readmissions. Readmissions due to any reason were higher in the exposed group, occurring in 44.3% (520 of 1173) of the exposed compared to 34.2% (712 of 2079) of the unexposed participants, with median time to readmission being shorter (12 and 20.5 days respectively) in the exposed group (*Table 2*). Readmission due to dehydration occurred in 7.7% of all participants (249 of 3252) and 20.2% of all readmissions (249 of 1232), accounting for 29.0% (151 of 520) of the readmissions in the exposed group compared to 13.8% (98 of 712) of the readmissions in the unexposed group, with comparable median time to readmission (10 *versus* 9 days). Total person time of the exposed group was 64 757 days and 130 907 days for the unexposed group, resulting in an incidence rate of 2.3 events/1000 days among exposed *versus* 0.7 events/1000 days among unexposed, yielding an IRR of 3.11 (95% c.i. 2.40 to 4.06).

**Table 2:** Number of events (at least one readmission due to any reason and dehydration or death respectively) within 90 days after index surgery among the 3252 analysed participants

	Total *N* = 3252	Exposed *N* = 1173	Unexposed *N* = 2079	*P*
Readmission due to any reason	1232 of 3252 (37.9)	520 of 1173 (44.3)	712 of 2079 (34.2)	<0.001
Readmission due to dehydration	249 of 3252 (7.7)	151 of 1173 (12.9)	98 of 2079 (4.7)	<0.001
Diagnosis of dehydration at readmission	249 of 1232 (20.2)	151 of 520 (29.0)	98 of 712 (13.8)	<0.001
ICD-10 code for volume depletion or electrolyte imbalance	217 of 249 (87.1)	126 of 151 (83.4)	91 of 98 (92.9)	<0.001
ICD10 code for acute renal failure	68 of 249 (27.3)	58 of 151 (38.4)	10 of 98 (10.2)	<0.001
90-day mortality	19 of 3253 (0.6)	6 of 1173 (0.5)	13 of 2079 (0.6)	0.813

Variables are reported as frequency (proportion). Exposed was defined as at least two dispensations of ACEI, ARB or diuretics within 365 days prior to index surgery. ACEI, angiotensin-converting enzyme inhibitors; ARB, angiotensin II receptor blockers.

### Kaplan–Meier graphs and Cox regression models

**Fig. 2:**
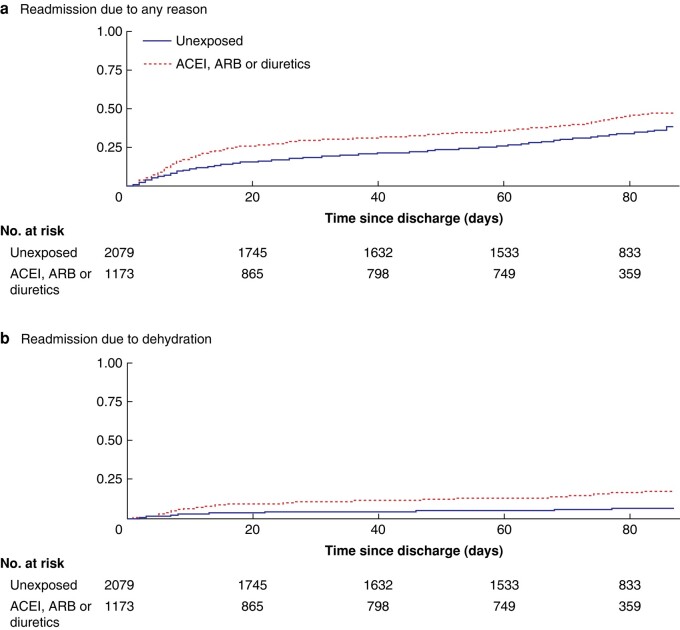
Kaplan–Meier curves on time to readmission due to any reason and dehydration respectively ACEI, angiotensin-converting enzyme inhibitors; ARB, angiotensin II receptor blockers.

The cumulative incidence proportion shows significant differences for exposed and unexposed groups regarding readmission due to dehydration as well as due to any reason (*P* < 0.001; *Fig. 2*). The HR ratio for readmission with diagnosis of dehydration, adjusted for age, gender, ASA score and CCI score, was 2.45 (95% c.i. 1.83 to 3.27; *Table 3*). Addition of treatment-related variables such as TNM stage, preoperative radiotherapy, perioperative bleeding, duration of index surgery and time between surgery and discharge during index admission changed the HR by less than 3.4% and therefore they were not included in the final model. The effect of dispensation of loperamide, opioids or oxycodone/naloxone was minimal and was not adjusted for. Diagnosis of cardiovascular disease or diabetes mellitus, and dispensation of calcium channel or beta blockers, were not included in the final model due to collinearity with the exposure to ACEI, ARB and diuretics. No significant effect modification was detected. In a subanalysis comparing different dispensations with unexposed, the adjusted HR was 3.51 for ACEI only, 3.34 for ACEI and diuretics combined, 2.04 for ARB and diuretics combined, 1.90 for ARB only and 1.22 for diuretics only (*Table 3*).

**Table 3:** Hazard rate ratios for exposure to preoperative use of ACEI, ARB or diuretics on the risk of 90-day readmission due to dehydration among the 3252 analysed participants

	Unadjusted			Adjusted*		
	HR	95% c.i.	*P*	HR	95% c.i.	*P*
Unexposed ACEI, ARB or diuretics	Ref 3.00	2.33,3.87	<0.001	Ref 2.45	1.83,3.27	<0.001
Unexposed ACEI only	Ref 3.92	2.81,5.46	<0.001	Ref 3.51	2.45,5.03	<0.001
Unexposed ACEI and diuretics combined	Ref 4.27	2.94,6.18	<0.001	Ref 3.34	2.22,5.03	<0.001
Unexposed ARB and diuretics combined	Ref 2.48	1.53,4.02	<0.001	Ref 2.04	1.24,3.37	0.005
Unexposed ARB only	Ref 2.04	1.25,3.33	0.005	Ref 1.90	1.14,3.17	0.013
Unexposed Diuretics only	Ref 1.86	1.10,3.16	0.021	Ref 1.22	0.70,2.15	0.481

ACEI, angiotensin-converting enzyme inhibitor; ARB, angiotensin II receptor blocker; Ref, Reference value 1.0. *Adjusted for categorical variables age, gender, score and Charlson Comorbidity Index score (weighted).

### Pilot study

In the record-based pilot study, exposure to ACEI or ARB for patients in the Stockholm region was 24.9% (179 of 718). In total, 30.2% (217 of 718) were readmitted at least once within 90 days after index surgery. Readmission due to any reason was higher in the exposed group, occurring in 38.5% (69 of 179) of the exposed compared to 27.5% (148 of 539) of the unexposed participants. Among all readmitted patients, 44.2% (96 of 217) had signs of dehydration or acute renal failure (based on ICD-10 codes or notes in medical records). Readmission due to dehydration accounted for 59.4% (41 of 69) of all readmissions in the exposed group, compared to 37.2% (55 of 148) in the unexposed group. Total person time was 9875 days for the exposed group and 33 937.5 days for the unexposed group, resulting in an incidence rate of 4.2 events/1000 days among exposed *versus* 1.6 events/1000 days among unexposed, yielding an IRR of 2.5 (95% c.i. 1.7 to 3.9).

## Discussion

This nationwide cohort study shows that patients with rectal cancer with preoperative use of ACEI, ARB and diuretics have a two- to three-fold risk of being readmitted due to dehydration within 90 days after elective anterior resection and ileostomy formation compared to unexposed participants.

In this study, 37.9% of the participants were readmitted within 90 days after index surgery with 20.2% of the remissions being due to dehydration. These findings give an incidence of dehydration-related readmissions after ileostomy formation of 7.7%, similar to the previously described 5.0%–10.3%^1,5,9^. Median time to readmission was 10 days, which also is in line with previous findings varying from 7 to 13 days^11,19–21^. Others have previously reported that postoperative use of diuretics is a significant risk factor for readmission due to dehydration after ileostomy formation (OR 2.44, 95% c.i. 1.5 to 3.8; *P* = 0.0001)^11^. Similar findings were observed by others; however, they were not statistically significant (*P* = 0.08)^22,23^. Another group previously reported usage of ACEI or ARB being associated with increased risk of readmission for dehydration following ileostomy formation (OR 13.56, 95% c.i. 3.54 to 51.92; *P* < 0.001)^12^. A similar experience also concluded that usage of ACEI and ARB was associated with postoperative renal decline 6 months after stoma closure^2^. However, these are all limited to retrospective single-site cohort studies with different study populations and varying definitions of exposure, outcome and follow-up time.

The strengths of this study include its population-based design with usage of register data with almost complete national coverage, minimizing the risk of selection bias as well as increasing the generalizability. Besides detailed information on patient-, tumour- and treatment-related characteristics in the SCRCR, additional information was retrieved from other high-quality national registers from the National Board of Welfare and Statistics Sweden due to the unique Personal Identification Number assigned to all Swedish residents. As a result, a large cohort was obtained with complete follow-up. The pilot study also made it possible to validate the results of the main study.

Exposure to ACEI, ARB or diuretics resulted as expected in an exposed group characterized by increased age and comorbidity. The study is limited by exposure and outcome being restricted to only register-based variables, in contrast to the pilot study where information in medical records was obtained manually. Classification of exposure was defined as two dispensations of ACEI, ARB or diuretics within a defined time period. However, it was not possible to track compliance and potential interruptions of these medications in the Prescribed Drug Register, especially in the early postoperative phase. We therefore make the assumption that participants exposed preoperatively also were exposed post-surgery, which is confirmed by the record-based pilot study where exposure status was identified in the discharge summary of the index surgery. A postoperative disruption of medication would result in a misclassification of exposure diluting the result. In addition, outcome was defined by ICD-10 codes set by discharging doctors. Diagnosis of dehydration can be difficult due to variations in diagnosis criteria (for example, clinical signs, laboratory findings, stoma output, urine production, etc.) and were potentially missed or misclassified. Dehydration may also lead to other diagnoses such as hypotension, syncope, fall injuries, unconsciousness, arrhythmias, etc. that we did not measure. Therefore, using ICD-10 codes from the Inpatient Register most likely limits the estimation of the true incidence of dehydration-related readmissions. Compared to the pilot study, the proportion of exposure was higher and readmission due to dehydration was lower in the register-based study. However, despite differences in methodology, the overestimation of exposure and underestimation of outcome do not seem to severely bias the study findings as the incidence rate ratios were similar. Adjustment for age, gender, ASA and CCI score reduced expected confounding and further treatment- or cancer-related factors did not change estimates substantially, indicating a low level of residual confounding. It is not surprising that preoperative use of ACEI, ARB or diuretics increases the risk of postoperative dehydration after ileostomy formation because it results in a physiological condition associated with loss of electrolytes and water in combination with drugs affecting the kidney’s ability to regulate the homeostasis. The negative effect of ACEI and ARB seems to be even more pronounced than the effect of diuretics and pathophysiologically similar effects can be expected after ileostomy formation in an emergency setting. The absolute risk of dehydration is most likely underestimated in the present study, which should be accounted for when generalizing the findings. Many patients experience high outputs from the ileostomy early in the postoperative phase with a spontaneous gradual decrease in output, but this may also be a clinical sign for dehydration. This implies a risk for delayed discovery of renal failure, especially in the case of limited adaptive capacity of the kidneys. In fact, the proportion of readmissions due to acute renal failure was 10 times higher among the exposed compared to unexposed, compared to twice as high for dehydration.

The finding of an overall readmission rate of almost 40% also confirms the problem with high rates of unplanned readmissions following rectal cancer surgery and ileostomy formation. Therefore, the ability to identify patients at risk for readmission is urgent in order to improve postoperative care and usage of healthcare resources. Swedish package leaflets do not mention specifically cautious use of ACEI, ARB or diuretics in the case of presence of an ileostomy. Length of hospital stay has also diminished after introduction of minimally invasive surgery and protocols for enhanced recovery. In consequence, adaptation to ileostomy occurs to a greater extent outside hospital care with restricted possibilities to monitor signs of dehydration. Enhanced awareness and improved postoperative follow-up may prevent severe dehydration, unnecessary readmissions as well as potentially prevent long-term effects on renal function.

Overall, preoperative use of ACEI, ARB or diuretics is associated with an increased risk for readmission due to dehydration in patients treated with anterior resection and ileostomy. In the majority of patients, readmission occurred within the first 2–3 weeks after discharge and might be avoided by enhanced awareness and closer postoperative follow-up.

## Supplementary Material

zrae051_Supplementary_Data

## Data Availability

The study is based on depersonalized data which cannot be shared by the authors due to the instructions of the ethical permission. For further questions regarding data availability, please contact the board of CRCBaSe (email: crcbase@mmk.ki.se).
