# Correction to: Preoperative use of angiotensin-converting enzyme inhibitors, angiotensin II receptor blockers and diuretics increases the risk of dehydration after ileostomy formation: population-based cohort study

**DOI:** 10.1093/bjsopen/zraf174

**Published:** 2026-01-01

This is a correction to: Louise de la Motte, Caroline Nordenvall, Anna Martling, Christian Buchli, Preoperative use of angiotensin-converting enzyme inhibitors, angiotensin II receptor blockers and diuretics increases the risk of dehydration after ileostomy formation: population-based cohort study, *BJS Open*, Volume 8, Issue 3, June 2024, zrae051, https://doi.org/10.1093/bjsopen/zrae051

In the **Abstract**, the reported Incidence Rate Ratio is wrong in the **Results** section. It should be: “3.11 (95% c.i. 2.40 to 4.06, *P* < 0.001)”. This error has been outlined only in this correction notice to preserve the version of record.

