# Supplementary material for: Preoperative use of angiotensin-converting enzyme inhibitors, angiotensin II receptor blockers and diuretics increases the risk of dehydration after ileostomy formation: population-based cohort study
Source: BJS Open. 2024 May 31;8(3):zrae051. doi: 10.1093/bjsopen/zrae051 (PMC11140823; doi:10.1093/bjsopen/zrae051)
Supplement: zrae051_Supplementary_Data [file zrae051_supplementary_data.docx]

**Preoperative use of Angiotensin converting enzyme inhibitors, Angiotensin II receptor blockers and diuretics increases the risk of dehydration after ileostomy formation:**

**a population-based cohort study**

Louise de la Motte^ab^, Caroline Nordenvall^ab^, Anna Martling^ab^, Christian Buchli^ab^

^a^ Department of Molecular Medicine and Surgery, Karolinska Institutet, Stockholm, Sweden

^b^ Department of Pelvic Cancer, Colorectal Surgery Unit, Karolinska University Hospital, Stockholm, Sweden

**Corresponding author.** Dr Louise de la Motte, Department of Molecular Medicine and Surgery, Karolinska Institutet, Karolinska University Hospital, Solna (L1:00), 171 76 Stockholm, Sweden. Email: [louise.de.la.motte@ki.se](mailto:louise.de.la.motte@ki.se)

| **Supplementary Appendixes** |  |
| --- | --- |
| Appendix 1 – Variables to define study population | *page 2* |
| Appendix 2 – Variables to define exposure  Appendix 3 – Variables to define outcome  Appendix 4 – Additional variables | *page 3*  *page 4*  *page 5+6* |
|  |  |

**Supplementary Appendixes**

| **Appendix 1. Variables used to define study population** | | |
| --- | --- | --- |
| **Variable value** | **Variable description** | **Type of Register** |
| Emergency_surgery | Emergency or elective surgery (1=elective/2=acute) | The Swedish Colorectal Cancer Register |
| Proceduredate | Date of surgery | The Swedish Colorectal Cancer Register |
| Surgery_type | Type of surgery (1-14, 7 = anterior resection) | The Swedish Colorectal Cancer Register |
| A2_skydd | Temporary loop ileostomy (1=Yes/0=No) | The Swedish Colorectal Cancer Register |
| Curative_proc | Curative procedure (1=Yes/0=No) | The Swedish Colorectal Cancer Register |
| Location | Tumor location (1=Colon, 2=Rectum) | The Swedish Colorectal Cancer Register |
| A2_preavl | Temporary loop ileostomy prior to index surgery (1=Yes/0=No) | The Swedish Colorectal Cancer Register |
| Mstage_clin | M stage, clinical (1=Yes/0=No) | The Swedish Colorectal Cancer Register |
| ICD10: N182, N183, N184, N185, N189 | Diagnosis of chronic renal failure (1=Yes/0=No) | Inpatient Register |
| ICD10: JFG00, JFG10, JFG20, JFG23, JFG26, JFG29, JFG30, JGF33, JFG36 | Diagnosis of stoma closure (1=Yes/0=No) | Inpatient Register |
| ICD10: JGB00, JGB01, JGB03, JGB04 | Diagnosis of anterior resection | Inpatient Register |
| ICD10: JFF10, JFF11, JFF13 | Diagnosis of ileostomy | Inpatient Register |
| Indate | Admission date | Inpatient Register |
| Outdate | Discharge date | Inpatient Register |
| Deathdate | Death date | Cause of Death Register |
| Migrationdate | Migration date | Statistics Sweden |
| Type | Type of migration (1=In/2=out) | Statistics Sweden |

Note: ICD10 = International Classification if Disease (ICD), 10^th^ edition.

| **Appendix 2. Variables used to define exposure** | | |
| --- | --- | --- |
| **Variable description** | **Variable definition** | **Type of Register** |
| Dispense date | edate | Prescribed Drug Register |
| Diuretics, only | ATC code: C03 | Prescribed Drug Register |
| Angiotensin converting enzyme inhibitors, only | ATC code: C09A | Prescribed Drug Register |
| Angiotensin converting enzyme inhibitors, combined | ATC code: C09B or C09A + C03 | Prescribed Drug Register |
| Angiotension II Receptor Blocker, only | ATC code: C09C | Prescribed Drug Register |
| Angiotension II Receptor Blocker, combined | ATC code: C09D or C09C + C03 | Prescribed Drug Register |

Note: ATC = Anatomical Therapeutical Chemical

| **Appendix 3. Variables used to define outcome** | | |
| --- | --- | --- |
| **Variable description** | **Variable Definition** | **Type of Register** |
| Dehydration | ICD-10 E86 Volume depletion | Inpatient Register |
| Electrolyte imbalance | ICD-10 E870 Hyperosmolality and hypernatremia  ICD-10 E871 Hypo-osmolality and hyponatremia  ICD-10 E875 Hyperkalemia  ICD-10 E876 Hypokalemia  ICD-10 E878 Other disorders of electrolyte and fluid balance, not elsewhere classified | Inpatient Register |
| Acute renal failure | ICD-10 N17 Acute renal failure  ICD-10 N19 Unspecified kidney failure  ICD-10 N990 Postprocedural renal failure | Inpatient Register |
| Death | Date of death | Cause of Death Register |

Note: ICD10 = International Classification if Disease (ICD), 10^th^ edition.

| **Appendix 4. Additional variables** | | |
| --- | --- | --- |
| **Variable name** | **Variable description** | **Type of Register** |
| Diagage | Age at CRC diagnosis (years) | Swedish Colorectal Cancer Register |
| Sex | Gender at diagnosis (1=male/2=female) | Swedish Colorectal Cancer Register |
| Height | Patient height at diagnosis (centimeter). | Swedish Colorectal Cancer Register |
| Weight | Patient weight at diagnosis (kg) | Swedish Colorectal Cancer Register |
| Asa_class | ASA classification (categorical, 1-5) | Swedish Colorectal Cancer Register |
| Neoadj_rt or neoadj_rt_ct | Preoperative Radiotherapy (1=Yes/0=No) | Swedish Colorectal Cancer Register |
| Surgery_dur | Length of index surgery (minutes) | Swedish Colorectal Cancer Register |
| Perop_bleed | Perioperative bleeding during index surgery (mililiter) | Swedish Colorectal Cancer Register |
| Tstage_path_cat | T stage, pathological (categorica, 1-6) | Swedish Colorectal Cancer Register |
| Nstage_path | N stage, pathological (categorical, 1-4) | Swedish Colorectal Cancer Register |
| Mstage_path | M stage, pathological (categorical, 1-3) | Swedish Colorectal Cancer Register |
| Adj_cyt | Postoperative chemotherapy (1=Yes/0=No) | Swedish Colorectal Cancer Register |
| ATC code: C08C, C08D, C08E, C08G | Calcium Channel Blockers (1=Yes/0=No) | Prescribed Drug Register |
| ATC code: C07A-F | Beta Blocking Agents (1=Yes/0=No) | Prescribed Drug Register |
| ATC code: N02A | Opioids, all (1=Yes/0=No) | Prescribed Drug Register |
| ATC code: N02AA55 | Opioids, oxycodone/naloxone only (1=Yes/0=No) | Prescribed Drug Register |
| ATC code: A07DA03 | Loperamide (1=Yes/0=No) | Prescribed Drug Register |
| ICD10: I00-99 | Diagnosis of cardiovascular disease (1=Yes/0=No) | Inpatient Register |
| ICD10: E10-14 | Diagnosis of diabetes mellitus (1=Yes/0=No) | Inpatient Register |
| Cci_weighted | CCI score (weighted) | Inpatient Register |
| Birthcountry | Country of Birth (1=Sweden/0=not Sweden) | Statistics Sweden |

Note: ASA = American Society of Anesthesiologists status, ATC = Anatomical Therapeutical Chemical, ICD10 = International Classification if Disease 10^th^ edition, CCI = Charlson Comorbidity Index.
